# Clinical correlates of diagnostic certainty in children and youths with Autistic Disorder

**DOI:** 10.1186/s13229-024-00592-7

**Published:** 2024-04-03

**Authors:** Eya-Mist Rødgaard, Borja Rodríguez-Herreros, Abderrahim Zeribi, Kristian Jensen, Valérie Courchesne, Elise Douard, David Gagnon, Guillaume Huguet, Sebastien Jacquemont, Laurent Mottron

**Affiliations:** 1 CIUSSS du Nord-de-l’Île-de-Montréal, Montréal, QC Canada; 2 Département de psychiatrie et addictologie, Université de Montréal, Montréal, QC Canada; 3 Service des Troubles du Spectre de l’Autisme et apparentés, Département de psychiatrie, Lausanne University Hospital (CHUV), Lausanne, Switzerland; 4 UHC Sainte-Justine Research Center, Université de Montréal, Montréal, QC Canada; 5 Department of Neurology and Neurosurgery, McGill University, Montréal, QC Canada

**Keywords:** Diagnosis, Certainty, ADOS, Macrocephaly

## Abstract

**Background:**

Clinicians diagnosing autism rely on diagnostic criteria and instruments in combination with an implicit knowledge based on clinical expertise of the specific signs and presentations associated with the condition. This implicit knowledge influences how diagnostic criteria are interpreted, but it cannot be directly observed. Instead, insight into clinicians’ understanding of autism can be gained by investigating their diagnostic certainty. Modest correlations between the certainty of an autism diagnosis and symptom load have been previously reported. Here, we investigated the associations of diagnostic certainty with specific items of the ADOS as well as other clinical features including head circumference.

**Methods:**

Phenotypic data from the Simons Simplex Collection was used to investigate clinical correlates of diagnostic certainty in individuals diagnosed with Autistic Disorder (*n* = 1511, age 4 to 18 years). Participants were stratified by the ADOS module used to evaluate them. We investigated how diagnostic certainty was associated with total ADOS scores, age, and ADOS module. We calculated the odds-ratios of being diagnosed with the highest possible certainty given the presence or absence of different signs during the ADOS evaluation. Associations between diagnostic certainty and other cognitive and clinical variables were also assessed.

**Results:**

In each ADOS module, some items showed a larger association with diagnostic certainty than others. Head circumference was significantly higher for individuals with the highest certainty rating across all three ADOS modules. In turn, head circumference was positively correlated with some of the ADOS items that were associated with diagnostic certainty, and was negatively correlated with verbal/nonverbal IQ ratio among those assessed with ADOS module 2.

**Limitations:**

The investigated cohort was heterogeneous, e.g. in terms of age, IQ, language level, and total ADOS score, which could impede the identification of associations that only exist in a subgroup of the population. The variability of the certainty ratings in the sample was low, limiting the power to identify potential associations with other variables. Additionally, the scoring of diagnostic certainty may vary between clinicians.

**Conclusion:**

Some ADOS items may better capture the signs that are most associated with clinicians’ implicit knowledge of Autistic Disorder. If replicated in future studies, new diagnostic instruments with differentiated weighting of signs may be needed to better reflect this, possibly resulting in better specificity in standardized assessments.

## Background

There are currently no known biomarkers for autism, and no firm biological or neurological definition of the underlying nature of the condition. Autism was initially formulated as a distinct condition based on clinical observations of children who exhibited differences in social and communicative functions as well as repetitive behaviours and interests [1]. Since then, attempts to codify the definition of autism have resulted in several iterations of diagnostic criteria [2]. However, the diagnostic criteria do not provide an unambiguous definition of autism since the codified criteria leave room for diverging interpretation. To apply the criteria in diagnosing autism, a clinician must have additional knowledge of the qualitative expression of different autism signs, or symptoms [3]. For example, some signs, such as abnormal eye-contact, are also associated with other conditions, but the qualitative characteristics of the abnormalities differ between the conditions and the clinician must recognize how and when the specific quality is associated with autism rather than another condition [3]. The clinician must also have knowledge of clinical thresholds for distinguishing autism signs from behaviours that are uncommon, but should be considered part of normal variation [4]. A clinician’s additional knowledge comes from experience and exposure, or in other words, from encountering autism in a clinical setting or from otherwise observing and interacting with individuals with autism as well as individuals with other distinct conditions. This knowledge gained from experience cannot be easily written down or directly transferred to another person and is thus a form of implicit (or tacit) knowledge [5]. The diagnostic practice of a clinician, i.e., who is and who is not diagnosed with autism, is guided by the codified diagnostic criteria, but these are modulated by the clinician’s implicit knowledge of and expertise in the condition.

Since clinicians’ implicit knowledge about autism cannot be fully captured in a formal definition, but rather is gained from interacting with autistic individuals, a circularity in the definition of autism arises: clinicians’ implicit knowledge influences their collective diagnostic practices which has a direct impact on the composition of the diagnosed autism population; the diagnosed population in turn forms the basis of who will be included in scientific studies about autism, and who will be seen as representatives of autism, which further contributes to clinicians’ implicit knowledge. Due to this circularity, clinicians’ implicit knowledge is central to shaping the concept of autism and to our understanding of the condition. Furthermore, the circularity can lead to changes in the understanding of autism over time even in the absence of changes to the diagnostic criteria. Whereas the explicit diagnostic criteria are codified in diagnostic manuals, the implicit knowledge cannot be observed directly. One way to gain insight into this is to question clinicians about their certainty of a given diagnosis and correlate this measure of certainty to observable characteristics in the individual being diagnosed, since the certainty of an autism diagnosis can be a measure of how closely the individual matches the clinician’s understanding of autism.

Previous studies have used diagnostic certainty, or confidence, e.g. as a proxy for symptom severity [6]. Certainty scores have also been used to investigate whether demographic characteristics such as age, sex, or parent income [7–9], cognitive or behavioural variables such as IQ or adaptive functioning [10–12], or genetic factors such as *de novo* mutations [13] are associated with the diagnostic certainty of an autism diagnosis. Studies tend to find no association between certainty rating and sex [7, 8], and findings have been mixed regarding the associations between certainty and variables such as adaptive or cognitive functioning, and age [8, 10–12]. Clinical assessment is generally guided by the quantification of symptom severity, using diagnostic instruments such as the Autism Diagnostic Observation Schedule (ADOS). Certainty of an autism diagnosis has been found to be significantly but modestly correlated with ADOS scores [11] and other measures of symptom severity [10, 12]. This means that a substantial number of individuals with a relatively high ADOS score are not necessarily diagnosed with the highest certainty, and conversely, that some individuals may be rated with the highest certainty despite having a relatively low ADOS score. An explanation for this could be that the quality of the symptoms that are present is more relevant for diagnostic certainty than the number of symptoms. Furthermore, some ADOS items may generally be more indicative of autism than others, and thus may be more strongly associated with diagnostic certainty. For example, the presence of a few highly specific signs with the right qualitative expression could therefore result in high diagnostic certainty despite a low total number of symptoms. As such, we aim to investigate how individual items in the ADOS are associated with clinicians’ certainty rating.

Another possible explanation for the modest association between certainty and symptom severity is that other factors, not directly represented in the ADOS, may also contribute to the clinician’s certainty. However, as mentioned above, previous studies have not shown consistent patterns of associations between certainty ratings and factors such as the level of cognitive or adaptive functioning [8, 10–12]. We wanted to further explore the associations of such factors as well as additional phenotypes such as language level and head circumference (HC). HC or other measures of brain size have been extensively investigated in autism with many studies finding larger heads or brains to be associated with autism [14]. A meta-analysis [15] investigated the percentage of autistic individuals with macrocephaly, which is defined as having a HC greater than the 97th percentile, and found that 15.7% of individuals with autism met this criterion compared to around 3% expected in the general population. Since macrocephaly during some period of development has been so strongly associated with autism in past research, this physical trait may directly or indirectly (i.e., by being related to other factors such as specific signs) impact the certainty of the clinicians performing autism assessments. We therefore wanted to further explore whether HC is associated with clinicians’ diagnostic certainty.

Many of the studies that have previously investigated diagnostic certainty have operationalized the construct as the level of confidence a clinician has that an individual is somewhere on the autism spectrum, which includes highly different phenotypic presentations, e.g. in terms of language ability (from fluent to no language) or IQ (from above or within the normal range to intellectual disability), thus introducing substantial heterogeneity. The term “the autisms” has previously been introduced to help explain the high heterogeneity, hypothesizing that multiple unknown but distinct subtypes exist within the autism clinical category that is now conceptualized as a spectrum [16]. If such subtypes exist, it is likely that each is associated with different symptom profiles, and that individuals with different types may all be recognised as being on the autism spectrum with high certainty, but for very different reasons. For example, in a child who meets DSM-IV criteria for Autistic Disorder, speech delay may be associated with high certainty that the child is on the autism spectrum. However, for a child who meets DSM-IV criteria for Asperger Syndrome, unusual but highly developed language may conversely contribute to high certainty that this child is on the autism spectrum [17]. Diagnostic certainty for autism spectrum disorder may thus reflect different, and sometimes opposite, deviations from typical behaviour, which is consistent with the wide heterogeneity that is accepted in the autism spectrum category. Investigations into correlates of certainty therefore run the risk of different effects negating each other resulting in an average that does not meaningfully capture why any particular included individual was diagnosed with high certainty.

To get insight into clinicians’ implicit knowledge through the investigation of diagnostic certainty, a better approach may be to focus on separate autism prototypes instead of the entire autism spectrum. A prototype represents a core presentation of a syndrome or condition, and individuals who are sufficiently similar to the prototype can be recognized by trained clinicians, presumably with higher certainty, the closer they are to the prototype [18–20].

In this study we investigate diagnostic certainty based on prototypical profiles by focusing on those individuals diagnosed with Autistic Disorder as per the DSM-IV. Although previous research has indicated problems with the validity of subgroups defined in the DSM-IV, the group of individuals diagnosed with Autistic Disorder is likely less heterogeneous, and thus more representative of a single prototype, compared to those diagnosed with any autism spectrum diagnosis (i.e. including Asperger Disorder, Pervasive Developmental Disorder Not Otherwise Specified (PDD-NOS) or DSM-5 Autism Spectrum Disorder). We may thus gain insight into which features are specifically associated with the Autistic Disorder prototype, and likely obtain a stronger signal for what is considered relevant for recognizing this Autistic Disorder prototype than had we included the whole spectrum. We utilize data from the Simons Simplex Collection, which has also been used in some previous studies that have included diagnostic certainty [7, 9, 13, 21]. These studies have examined diagnostic certainty for all individuals who have been diagnosed with an autism spectrum diagnosis, whereas we focus on those diagnosed specifically with Autistic Disorder.

### Aim

In the present study, we aimed to identify the specific clinical correlates of high certainty of an Autistic Disorder diagnosis. Therefore, we investigated the following research questions: does certainty correlate with total symptom load, are there specific ADOS items that are more highly associated with certainty than others, and are other variables such as proband demographics, HC, IQ, and language level associated with certainty.

## Methods

### Participants

The participants included in this study are part of the Simons Simplex Collection (SSC) [22]. The SSC is a database that contains behavioural, cognitive, and genetic data from approximately 2,800 individuals meeting the criteria for an autism diagnosis. Probands’ phenotypes were evaluated with a battery of instruments for which the descriptions are available on the Simons Foundation Autism Research Initiative website (https://sfari.org). The SSC only includes simplex cases and enrolment to the database is based on referral from clinical genetic centres, testing laboratories, web-based networks, or active online registration. The inclusion criteria for entry into the SSC database required probands to: (1) be between four and 18 years of age; (2) meet the criteria for a diagnosis of autism, Asperger syndrome, or autism spectrum; and (3) have a nonverbal mental age of at least 18 months. Only individuals who received a diagnosis of “Autism” or “Autistic Disorder” were included (*n* = 1511), i.e. those with a diagnosis of PDD-NOS, Asperger Disorder, or Autism Spectrum Disorder were not included. All analyses were conducted separately for those assessed with each ADOS module. Only a relatively small number of individuals were assessed with module 4 (*n* = 74 individuals, of whom 33 were diagnosed with autistic disorder), so this module was therefore not included. The demographics of the participants separated based on language level as measured by the ADOS module are presented in Table 1.

**Table 1:** Summary statistics of the included participants separated by the ADOS module used for assessment. The rows “Verbal IQ” and “Nonverbal IQ” show mean IQ values based only on deviation scores since ratio scores were not included in the analyses. The row “Highest certainty” shows the number and percentage of individuals who were diagnosed with Autistic Disorder with the highest possible certainty score. The rows “Father bachelor’s degree” and “Mother bachelor’s degree” show the numbers and percentages of individuals whose fathers and mothers, respectively, have obtained bachelor’s degrees

	ADOS Module 1	ADOS Module 2	ADOS Module 3
Total *n = 1511*	*n* = 394	*n* = 412	*n* = 705
Females	56 (14.2%)	68 (16.5%)	77 (10.9%)
Mean Age (SD)	8.0 (3.5)	7.5 (3.2)	9.9 (3.2)
Race	65% white, 8% Asian, 7% African American, 20% other	72% white, 5% Asian, 7% African American, 16% other	81% white, 3% Asian, 2% African American, 14% other
Ethnicity	16% Hispanic	15% Hispanic	11% Hispanic
Verbal IQ (SD)	57.9 (14.9) *n* = 102	76.6 (16.2) *n* = 301	89.4 (19.3) *n* = 690
Nonverbal IQ (SD)	70.9 (17.2) *n* = 152	83.9 (17.1) *n* = 347	93.1 (18.0) *n* = 695
Highest Certainty	329 (83.5%)	302 (73.3%)	401 (56.9%)
Father Bachelor’s Degree	221 (56.1%)	255 (61.9%)	407 (57.7%)
Mother Bachelor’s Degree	216 (54.8%)	249 (60.4%)	425 (60.3%)

### Measures

The SSC dataset contains many phenotypic variables based on different instruments. We have analysed a subset of these in this study. Since not all variables are available for all individuals, and in order to use as large a dataset as possible, we focused on the variables that were available for all or most of the individuals in the SSC, that are commonly used measures within autism research, and that represent physical or behavioral characteristics which could be observable to the clinician. These include autism symptomatology as measured by the ADOS, adaptive behaviours as measured by the Vineland Adaptive Behaviours Scale, internalizing and externalizing behaviours as measured by the Child Behaviour Checklist, and IQ. Furthermore, we included head circumference (HC), as this has been extensively researched and continuously associated with autism in previous research.

#### Verbal and non-verbal IQ

The SSC database contains IQ data based on several different instruments including the Differential Ability Scales 2nd edition (DAS-II), the Wechsler Intelligence Scale for Children 4th edition (WISC-IV), and the Mullen Scales of Early Learning (MSEL). Most of the IQ scores were calculated as deviation IQ, i.e. using an age-adjusted normed data set with a mean of 100 and a standard deviation of 15. A smaller set of the scores were calculated as ratio IQ by using normed data to estimate an age equivalent and dividing by the chronological age. Since these two methods produce scores that may not be fully comparable [23], we only included scores calculated with the deviation method in our analysis. Mean values of these are shown in Table 1. Ratio IQ scores tended to be lower than deviation IQ scores. The mean FSIQ for ratio and deviation scores combined were 42.2, 72.4, and 90.5 for those assessed with ADOS modules 1, 2, and 3, respectively.

#### Normalized head circumference

Head circumference (HC) was measured using a non-stretchable tape measure by measuring the widest part of the head. HC is strongly associated with factors such as sex, age, height, and ancestry. Therefore, a normalized head circumference variable was calculated to control for these variables. This was done as described by Chaste et al. [24] by using the data of all autistic individuals in the SSC to fit a linear model with HC as the dependent variable and height, weight, age, sex, and genetic ancestry information as independent variables. The normalized HC variable was defined as the residuals from the linear model, i.e. the difference between each individual’s measured HC and the expected HC based on the predictor variables.

#### Certainty variable

**Fig. 1:**
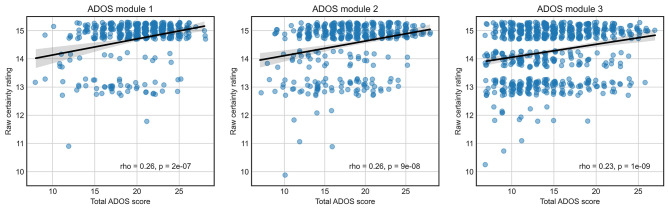
Correlations between the Total ADOS Score and Clinicians’ Certainty Rating. Each blue point represents an individual diagnosed with autism and deemed by the clinician to meet the DSM-IV-TR Autistic Disorder criteria. The black line indicates the best linear fit. As both the total ADOS score and certainty rating can only take integer values, the points were shifted by small random values (jitter) to ease visual inspection of the correlation

The SSC database contains a variable describing the certainty of the diagnosing clinician that the child has autism. The compilation of this variable is described in the SSC phenotypic data definition. If the child is deemed to lie somewhere on the autism spectrum, a base level of 5 points is given on the certainty variable. The clinician is then asked to rate how certain they are that the child meets the criteria for an autism spectrum diagnosis on a scale from 1 to 5, and this number is added to the certainty score. Additionally, if the child is deemed to meet the strict DSM-IV-TR criteria for autism, the clinician must again rate how certain they are of this diagnosis on a scale from 1 to 5, and this number is also added to the certainty score. Therefore, for children who receive an autism spectrum diagnosis, but do not meet the criteria for a DSM-IV-TR autism diagnosis, the certainty score has a minimum value of 6 and a maximum value of 10. For children who do meet criteria for DSM-IV TR autism, the certainty variable has a maximum value of 15 and a theoretical minimum value of 7 (even if the individual is deemed to meet the criteria for DSM-IV-TR autism, the clinician is allowed to rate a certainty of 1 for being on the spectrum and a certainty of 1 for meeting the DSM-IV-TR autism criteria, resulting in a total of 7) [22]. The certainty variable is thus not directly comparable between these two groups. In this study, only those who met the strict DSM-IV-TR criteria for Autistic Disorder were included. Among these, the certainty score (which can theoretically range from 7 to 15), was highly skewed with a large proportion of scores having the maximum certainty score of 15, and a decreasing number of individuals with progressively lower scores (Fig. 1). None of the children with a diagnosis of Autistic Disorder had a certainty score below 10. For most of the statistical analyses (see below), instead of using the raw certainty score from the SSC, the certainty scores were converted into a binary categorical variable: a certainty rating was coded as 1 if the score was 15 and certainty scores lower than 15 were coded as 0. This resulted in a less skewed distribution where the number of individuals with and without the highest possible certainty score were closer to each other.

#### Autism symptomatology

Total scores and individual item scores from the ADOS [25] were used as measures of symptom severity as well as symptom presentation. The ADOS module used to assess a participant was used as a proxy of language level: module 1 indicates no phrase speech, module 2 indicates phrase but not fluent speech, while module 3 indicates fluent speech. The ADOS was chosen since it is based on clinicians’ direct observations, and thus expected to correspond more closely to factors that determine certainty than, for example, the Autism Diagnostic Interview (ADI).

#### Vineland and CBCL measures

The Vineland Adaptive Behaviour Scale II was used as a measure of adaptive abilities. Externalizing and internalizing composite scales from the Child Behavior Checklist (CBCL) were also included in our analysis.

### Statistical analyses

#### Association between certainty and total Autism symptom score

Spearman correlation coefficients were calculated between the total ADOS score and the raw certainty score (from 1 to 15). The correlations were calculated separately for each ADOS module.

#### Association between certainty and individual ADOS items

Each ADOS item was converted to a binary variable representing the presence/absence of a clear presentation of a given sign. Thus, ADOS original item scores of zero and one were recoded as a zero, i.e. absence, and original item scores of two and three were recoded as a one, i.e. presence. For each ADOS item, the association between the binary coding and the binary certainty variable was investigated by calculating odds-ratios. Odds-ratios greater than one indicate that the presence of a given sign is associated with a higher likelihood of being diagnosed with the highest certainty. An odds-ratios less than one indicates a negative association where the presence of a sign is associated with a lower likelihood of being diagnosed with the highest certainty. The *scipy* Python package was used to calculate *p*-values and 95% confidence intervals for the odds-ratios. The analyses were performed separately for each ADOS module, and within each ADOS module, *p*-values for each ADOS item were corrected for multiple testing using the Benjamini-Hochberg method [26]. For some items, there were no individuals in one of the two certainty groups having an original item score of two or three, thus leading to division by zero when calculating the odds-ratios. In these cases, the odds ratio was instead estimated using the Haldane-Anscombe correction [27], by adding 0.5 to each of the counts used to calculate the odds-ratio.

#### Association between certainty and language level

The ADOS module with which an individual was evaluated was used as a proxy for the individual’s language level at the time of assessment. Binomial regression was used to investigate the association between language level and certainty. The binary certainty variable was the dependent variable and the ADOS module was the independent variable. Since the degree to which a given language level is normal or abnormal depends on age, age was also included as an independent variable, as well as an interaction term between age and the ADOS module. Binomial regression was performed using the ‘*glm’* function in R and the statistical significance of the effects was evaluated using the ‘*Anova’* function from the *car* package.

#### Association between diagnostic certainty and IQ, head circumference, and internalizing, externalizing, and adaptive behaviours

The associations between certainty and each of the following variables: verbal IQ, nonverbal IQ, the verbal/nonverbal IQ ratio, internalizing behaviour, externalizing behaviour, adaptive behaviour, and normalized HC, were assessed by investigating the group-level difference for each variable between those who were diagnosed with the highest certainty compared to those who were not. This was performed separately for each ADOS module as language level was found to influence certainty. Statistical significance of the group differences was evaluated using t-tests.

#### Association between normalized HC and other variables

Pearson correlation coefficients were calculated for each ADOS module between normalized HC and ADOS items as well as the binary diagnostic certainty, verbal IQ, nonverbal IQ, and verbal/nonverbal IQ ratio.

## Results

### Association between certainty and total Autism symptom score

We first investigated how well the certainty rating correlated with the ADOS total scores. For ADOS modules 1, 2, and 3 the correlations were 0.26 (*p* = 2e-7), 0.26 (*p* = 9e-8), and 0.23 (*p* = 1e-9), respectively (Fig. 1). Although this shows that certainty significantly correlated with total symptom load, the correlation was modest. There was thus a substantial proportion of participants with relatively low ADOS scores who were diagnosed with the highest certainty, and conversely, participants who did not receive the highest certainty rating despite having a relatively high ADOS score.

### Association between certainty and individual ADOS items

**Fig. 2:**
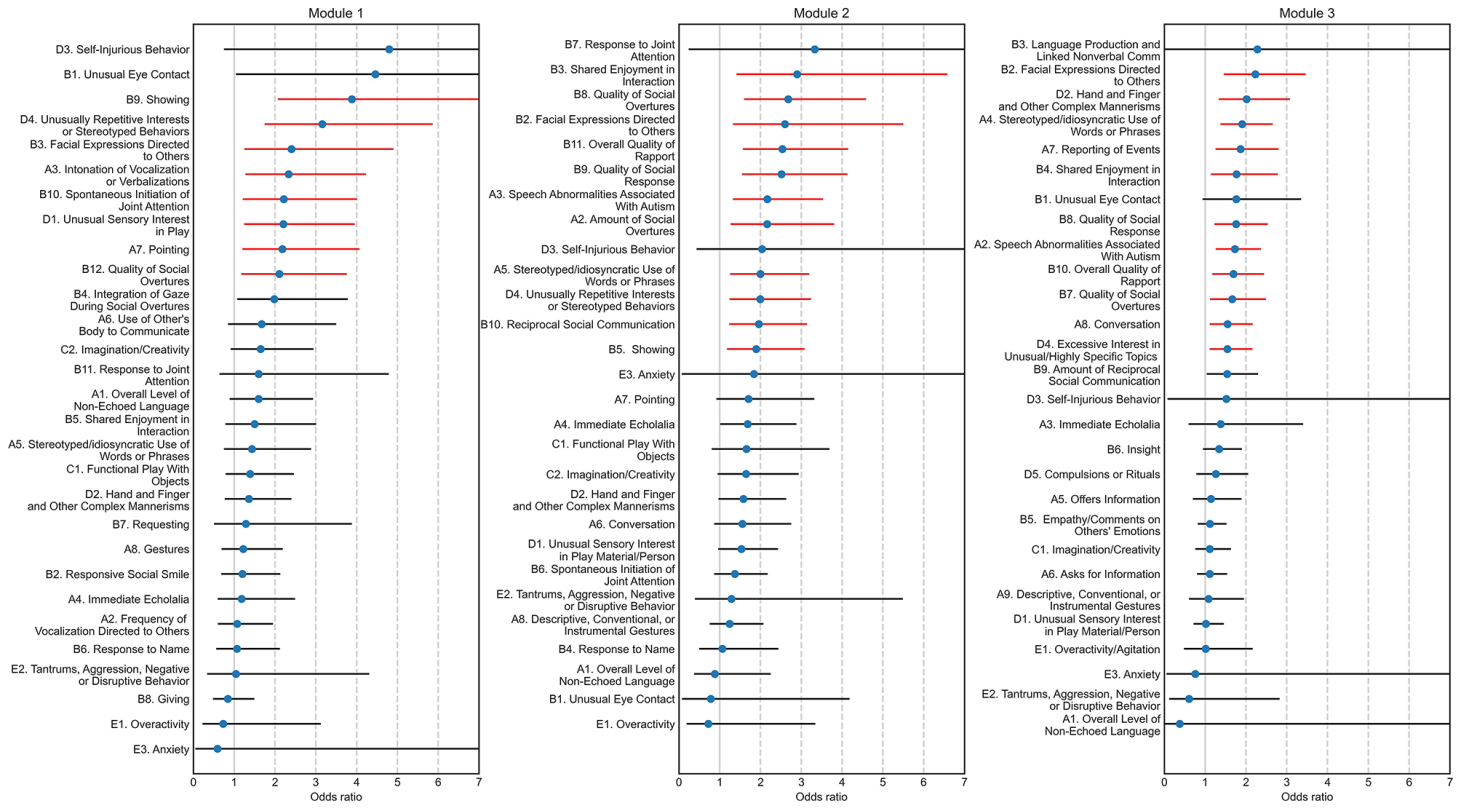
Odds-ratios of ADOS items and certainty. Forest plots showing the odds-ratios of the categorical certainty variable for each ADOS item in each of the three modules. An odds-ratio of 1 corresponds to no association, whereas an odds-ratio greater than 1 indicates that an individual with a given ADOS sign has a higher chance of being diagnosed with the highest certainty than an individual without the sign. The error bars indicate the 95% confidence intervals. Error bars in red indicate that the odds-ratio is statistically significantly different from 1 after correcting for multiple testing using the Benjamini-Hochberg method

To identify whether some ADOS items were more associated with diagnostic certainty than others, odds ratios for certainty were calculated for each item. These are shown for ADOS modules 1, 2, and 3 in Fig. 2. We found certain items to be associated with a prominent increase in the odds of having the highest certainty, whereas other signs showed no association or even had a small negative association. In both modules 1 and 3, the top five significant items most highly associated with an increased risk of having the highest certainty were characterized by a mix of items covering different symptom domains. However, for module 2, the top five items were within the social interaction domain. Within the communication domain, more items were significantly associated with certainty in modules 2 and 3 than in module 1.

### Association between certainty and language level

**Fig. 3:**
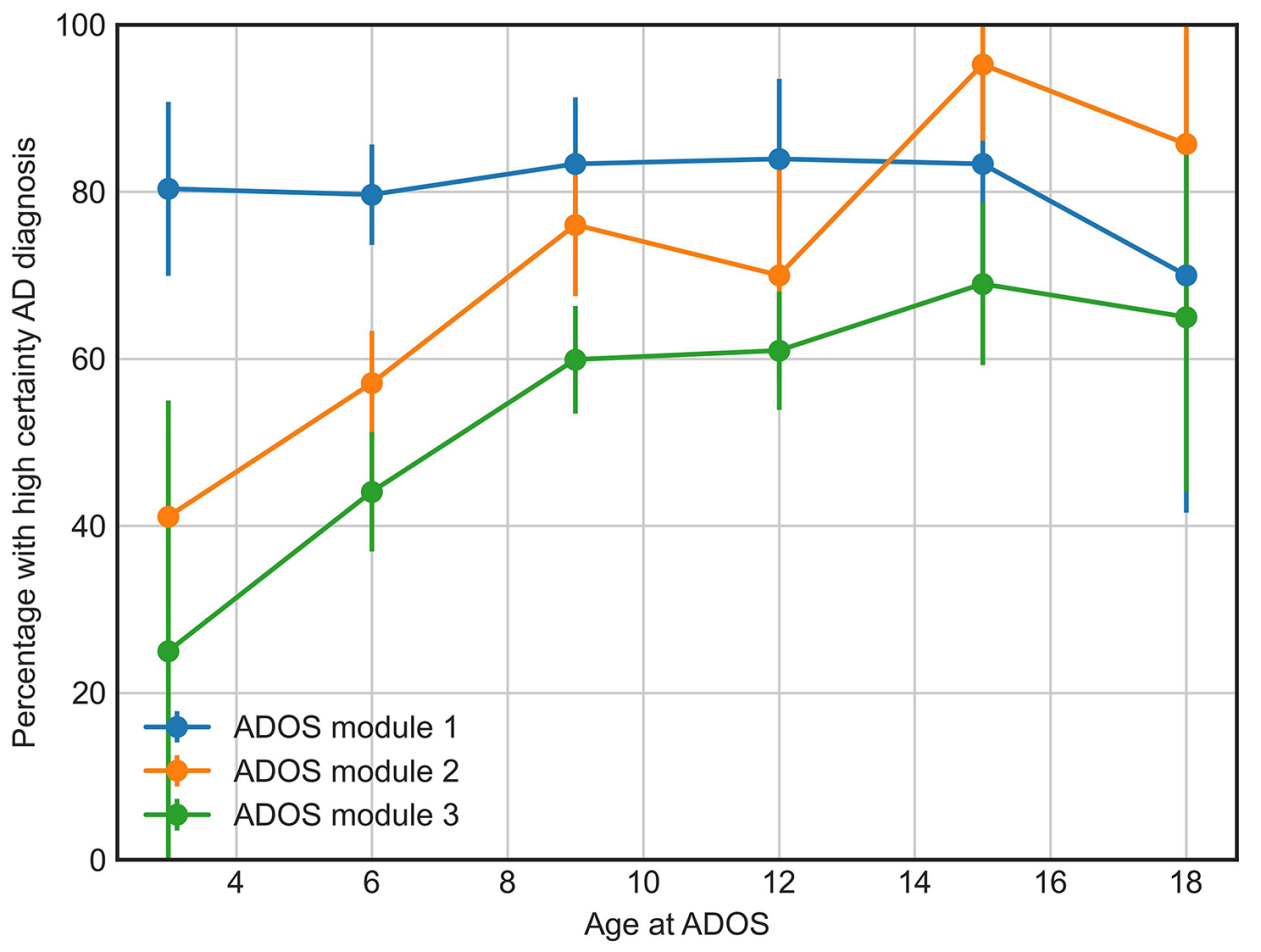
Diagnostic certainty by age and language level. Percentage of individuals being diagnosed with the highest certainty at different ages, among those assessed with each of the ADOS modules. For visual simplicity, participants were grouped into age brackets of 3 years, centered at 3, 6, 9, 12, 15, and 18 years old

In addition to the association between certainty and individual ADOS items, we also assessed how certainty was associated with language level. A child’s language level is used to determine which ADOS module to use for diagnostic assessment. Thus, the influence of language level was investigated by examining differences in diagnostic certainty between the ADOS modules. Age was also included as the association of language level and certainty was expected to vary between age groups. Using binomial regression, a significant interaction effect of ADOS module and age was found (*p* = 3e-9). Figure 3 shows the percentage of the individuals diagnosed with the highest certainty score across ages separately for ADOS modules 1 to 3. Among those evaluated with module 1, a large proportion, around 80%, of individuals were diagnosed with the highest certainty for all ages. In contrast, the proportion of those diagnosed with the highest certainty was significantly lower for those who were assessed with modules 2 and 3 at younger ages, but gradually increased at older ages. We further investigated whether diagnostic certainty differed according to sex or reported race and ethnicity. These three factors were tested individually while controlling for the effects of the ADOS module and age. No significant effects of sex, race, or ethnicity were found.

### Associations between certainty and IQ and internalizing, externalizing, and adaptive behaviours

**Fig. 4:**
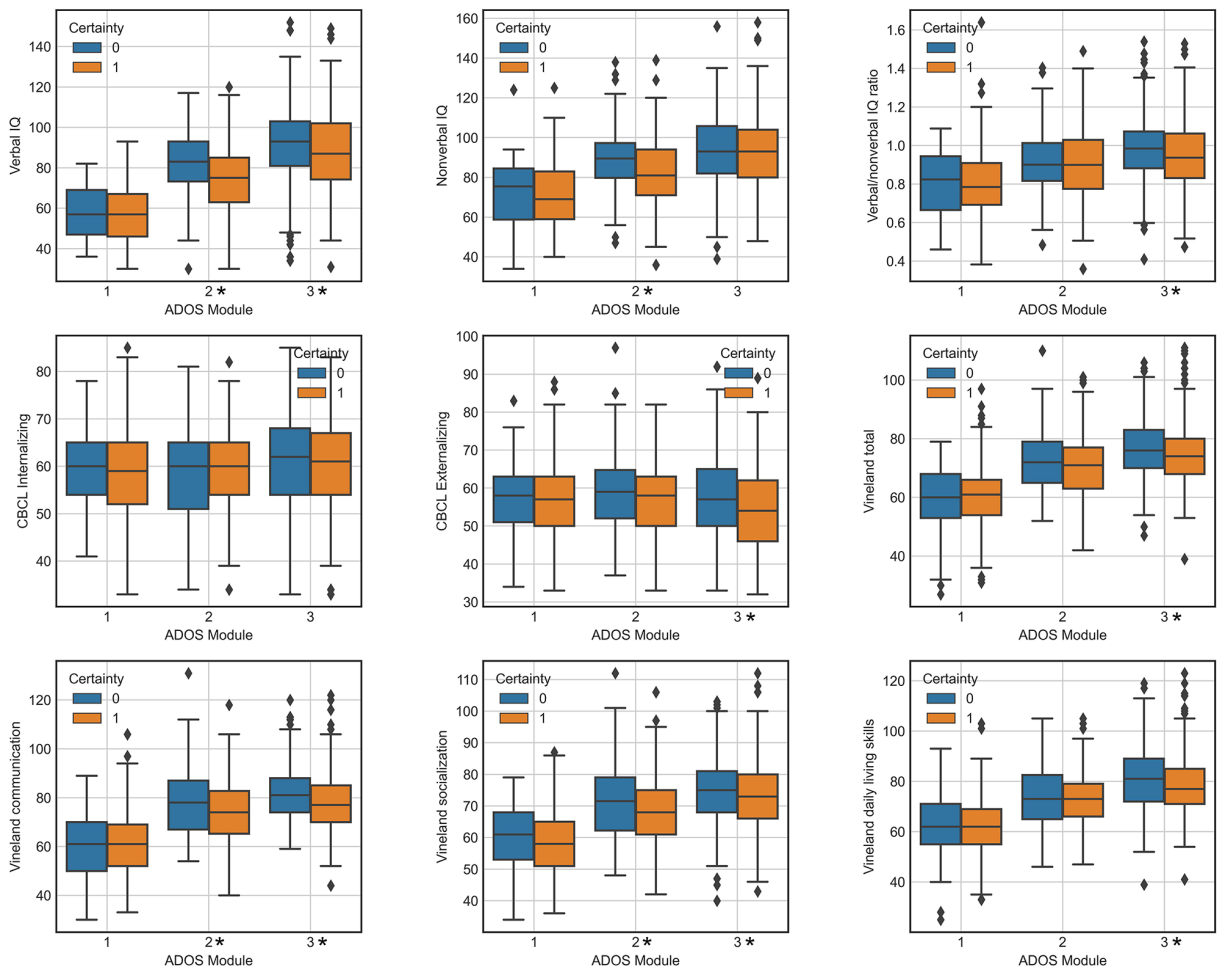
Associations between certainty and IQ and internalizing, externalizing, and adaptive behaviours. Boxplots showing the distributions of verbal IQ, nonverbal IQ, verbal/nonverbal IQ ratio, internalizing behaviour, externalizing behaviour, Vineland total score, and the scores for the three Vineland subscales of communication, socialization, and daily living skills. The orange boxes indicate those diagnosed with the highest certainty (15) whereas the blue boxes indicate those diagnosed with a lower certainty (< 15). Asterisks indicate statistically significant differences between those who were diagnosed with the highest certainty and those who were not

Associations between certainty and verbal IQ, nonverbal IQ, verbal/nonverbal IQ ratio, internalizing and externalizing behaviour as measured by the CBCL, and adaptive behaviours as measured by the Vineland scale were investigated. As diagnostic certainty was strongly associated with language level, the analyses were conducted separately by ADOS modules. The distributions of the variables for those who were diagnosed with the highest certainty, and those who were not, are presented in Fig. 4. We found a significant difference in verbal IQ between these groups of individuals assessed with ADOS modules 2 (t = -4.21, *p* = 3e-5) and 3 (t = -3.26, *p* = 0.001). Nonverbal IQ was significantly different between the high and low certainty groups in ADOS module 2 (t = -3.81, *p* = 2e-4), and the verbal/nonverbal IQ ratio was significantly different between the high and low certainty groups in ADOS module 3 (t = -2.78, *p* = 0.006). There were no differences in internalizing behaviours in any of the ADOS modules. Among those assessed with module 3, those diagnosed with the highest certainty had significantly lower externalizing behaviours (t = -3.15, *p* = 0.001), Vineland total scores (t = -2.58, *p* = 0.01), Vineland communication (t = -3.29, *p* = 0.001), Vineland socialization (t = -2.37, *p* = 0.02) and Vineland daily living skills (t = -2.32, *p* = 0.02) than those diagnosed with lower certainty. Among those assessed with module 2, individuals diagnosed with the highest certainty had significantly lower Vineland communication scores (t = -2.85, *p* = 0.005) and Vineland socialization scores (t = -2.22, *p* = 0.03) than those diagnosed with lower certainty.

### Association between certainty and head circumference

**Fig. 5:**
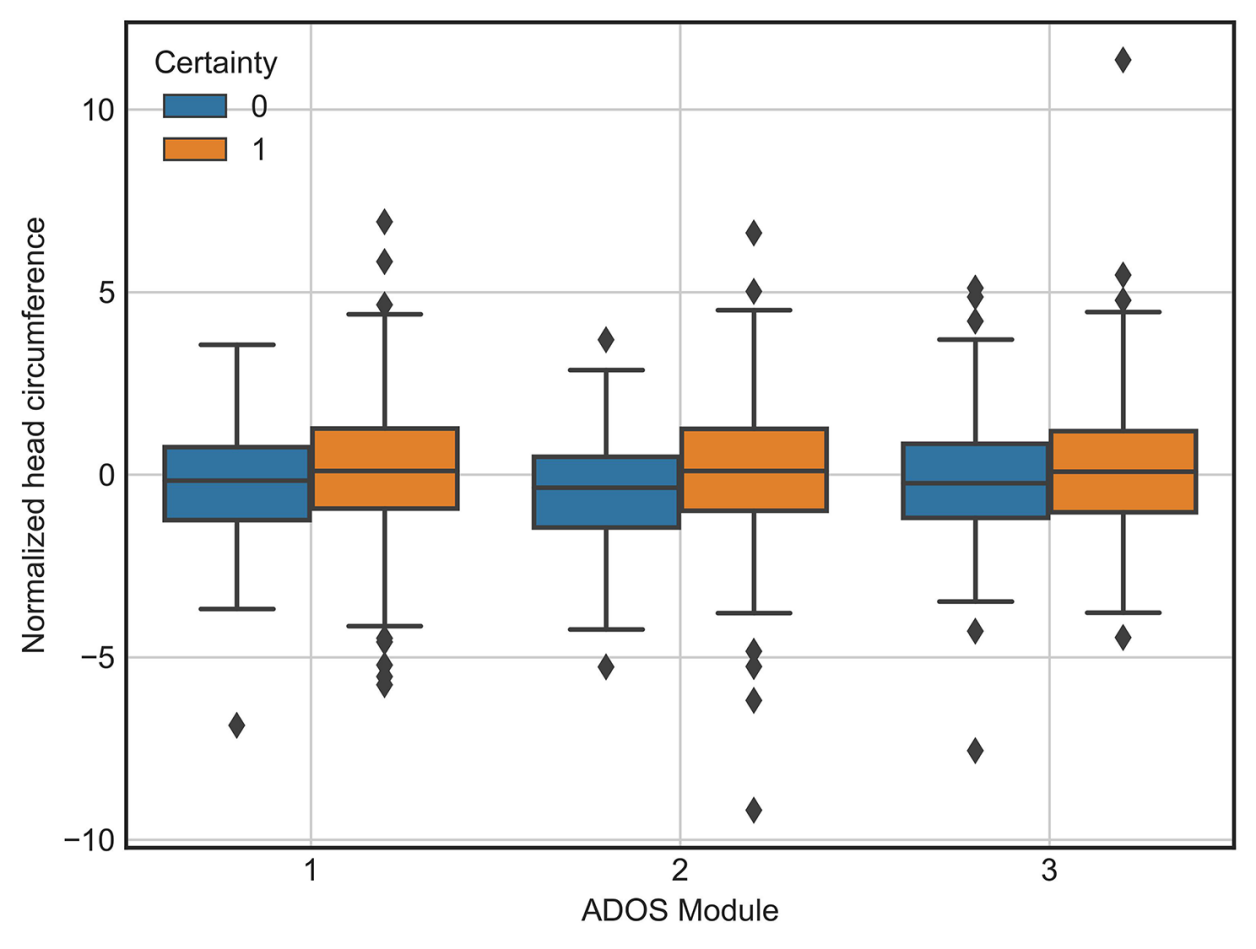
Association between certainty and head circumference. Boxplot showing the distribution of normalized head circumference for each of the ADOS modules. The orange boxes indicate those diagnosed with the highest certainty (15) whereas the blue boxes indicate those diagnosed with a lower certainty (< 15). Normalized head circumference was significantly different between the groups for all three ADOS modules

Across all three ADOS modules, normalized HC was significantly larger in those who were diagnosed with the highest certainty compared to those who were not (module 1: t = 2.17, *p* = 0.03; module 2: t = 3.17, *p* = 0.002; module 3: t = 2.03, *p* = 0.04) (Fig. 5). Among the 2.5% of the sample who had the highest normalized HC (the cutoff used for classification of macrocephaly in the general population), 85% were diagnosed with the highest certainty, whereas this was true for only 64% of the remaining participants.

### Correlation between normalized head circumference and other variables

**Fig. 6:**
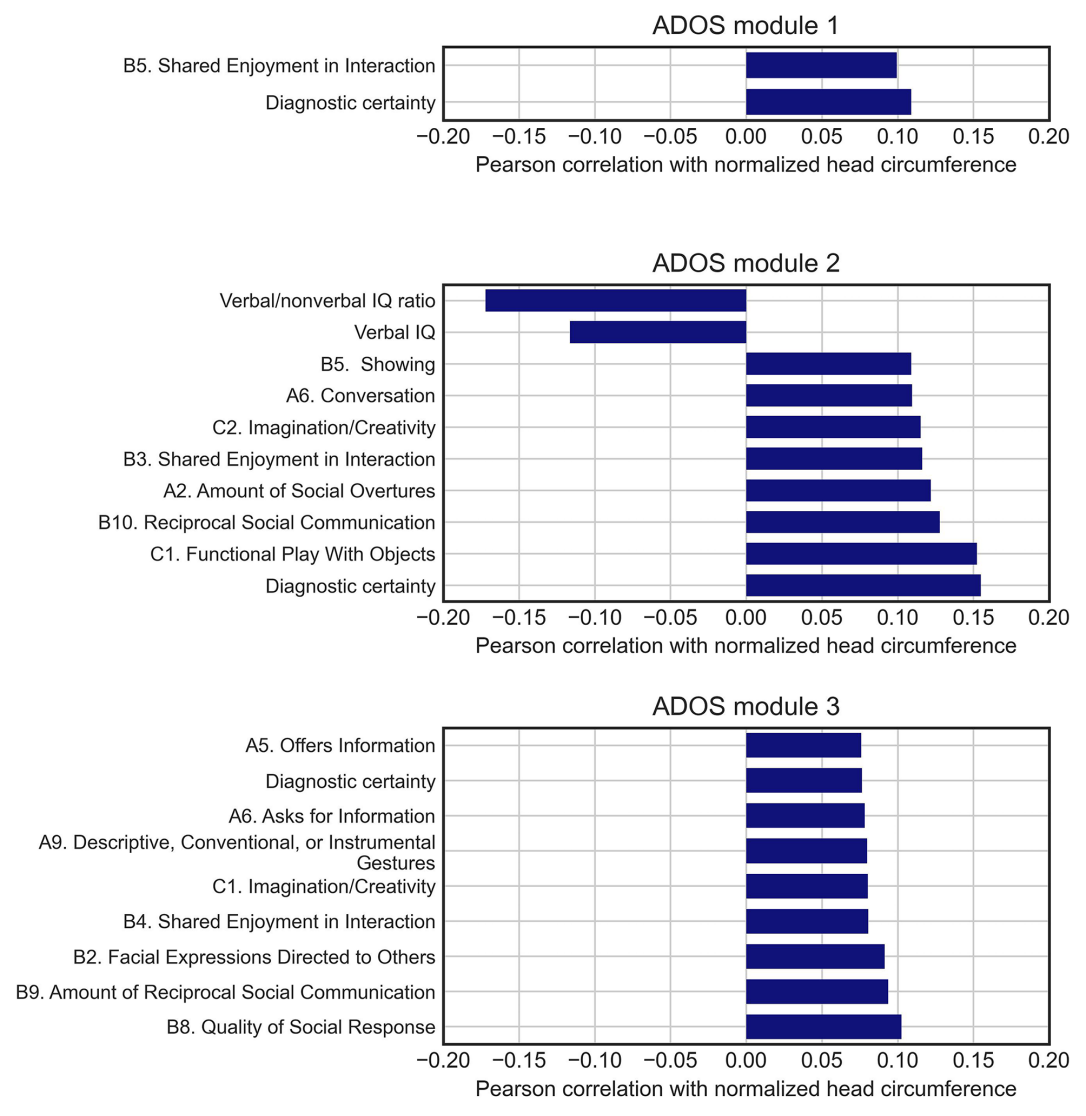
Correlation between normalized head circumference and other variables. Pearson correlation coefficients of different items and variables with normalized HC among those assessed with each of the three ADOS modules. Only variables and items with statistically significant correlations with normalized HC are shown in the figures

**Fig. 7:**
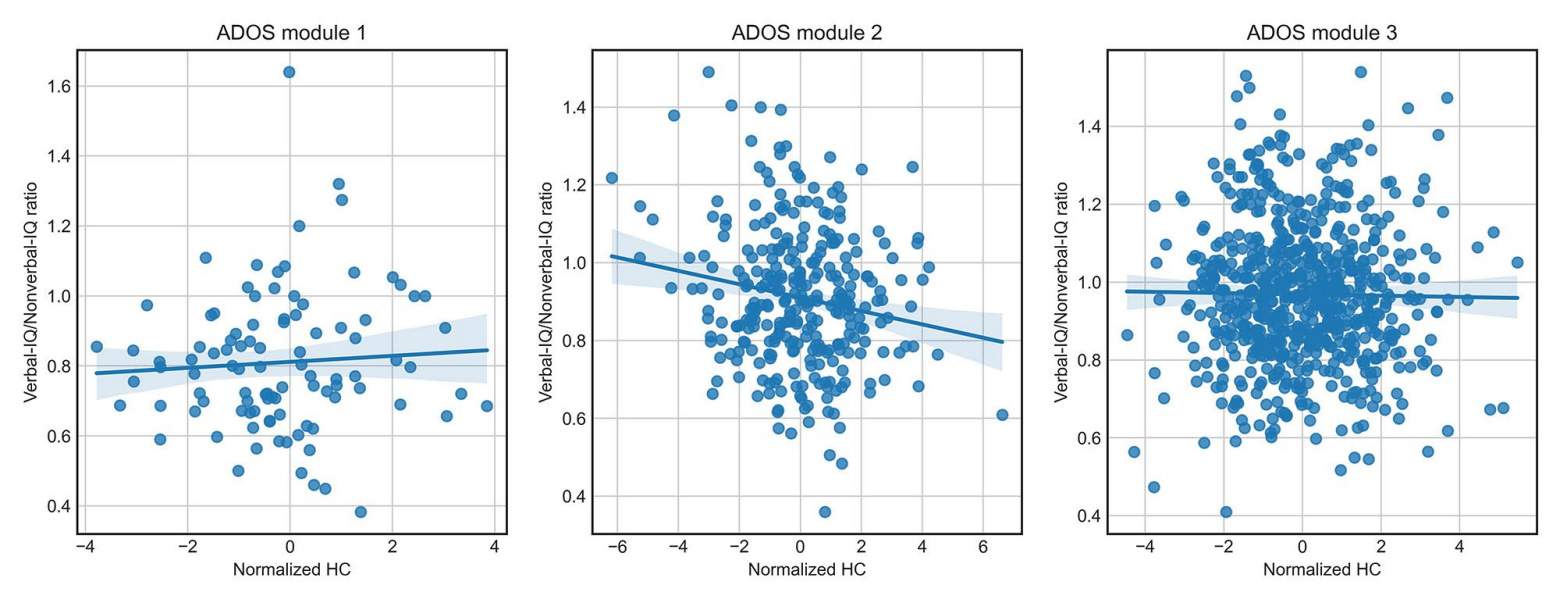
Correlation between normalized head circumference and verbal/nonverbal IQ ratio. Scatter plots showing the correlations between normalized head circumference and verbal/nonverbal IQ ratio among those assessed with ADOS modules 1, 2, and 3

Normalized HC was associated with diagnostic certainty for all ADOS modules. It is possible that clinicians directly observe an increased HC and that this leads to higher certainty, but since the HC increase associated with autism may not always be pronounced enough to be directly noticeable [15], it is possible that HC simply correlates with other characteristics, which the clinician associate with autism with high certainty. We therefore further investigated other correlates of HC such as ADOS items, certainty, verbal IQ, nonverbal IQ, and verbal/nonverbal IQ ratio. The Pearson correlation coefficients that were statistically significant are shown in Fig. 6. The ADOS item “Shared Enjoyment in Interaction” was significantly associated with HC for all three modules. No other items, except for “Shared Enjoyment in Interaction” and certainty, were significantly correlated with normalized HC in module 1. Other items that significantly correlated with HC in module 2 and 3 included signs associated with the social interaction, communication, and play behavioural domains. The strongest effect found was a negative correlation between normalized HC and the verbal/nonverbal IQ ratio among those assessed with ADOS module 2 (*r* = -0.17, *p* = 3e-3, Fig. 7). The IQ ratio did not correlate significantly with normalized HC in the two other modules.

## Discussion

The objective of this study was to explore factors and signs associated with clinicians’ certainty of an Autistic Disorder diagnosis to gain insight into the implicit knowledge that influences a clinician’s interpretation of diagnostic criteria and clinical decision making in general.

### Certainty and ADOS items

As expected from previous research [9–12], we found a modest correlation between diagnostic certainty and autism symptomatology, confirming that a substantial fraction of participants with a relatively low ADOS score were diagnosed with the highest certainty, whereas, some participants did not receive the highest certainty rating despite having relatively high ADOS scores. As mentioned previously, the modest correlation may be explained by different ADOS items having different associations with certainty such that some items contributing to the total score have little association with certainty. By investigating the associations between individual ADOS items and certainty, we confirmed that certain autism signs markedly increased the odds of being diagnosed with Autistic Disorder with the highest certainty, whereas some signs showed only minor associations with certainty, and others even showed a trend towards a negative association. This finding could suggest that particular signs have a stronger impact on how certain clinicians are in their diagnostic decision, likely reflecting that these characteristics are consistent with how the clinicians expect Autistic Disorder to appear. More items from the communication domain were significantly associated with certainty in ADOS modules corresponding to a higher level of language ability. This makes intuitive sense, as lower language ability in itself was found to be strongly associated with higher diagnostic certainty. In these individuals, the qualitative characteristics of language use are likely less important, whereas in individuals with more developed language abilities, qualitative atypicalities may have a larger influence on certainty.

The observation that some ADOS items are more associated with certainty than others may suggest that new ways of constructing assessment instruments could be investigated in the future to improve the specificity of the recognition-definition-investigation cycle [19]. Additionally, scores on instruments such as the ADOS are traditionally based on an equal weighting of all or some items [28] meaning that each included item equally contributes to the severity score. Some scoring algorithms (e.g., the calibrated severity score) that only include a select subset of all items have been found to identify autism with higher specificity [29]. However, given that different items may have different associations with recognizable manifestations of autism, it is also worth considering alternative algorithms with differential weighting of items. Furthermore, it is still an open question as to whether there are interactions between different signs which could improve discrimination; for example, the presence of two items together may have a higher weight than the sum of each item presented separately.

Such considerations may be particularly relevant in relation to the specificity of an instrument since individuals with other conditions may display a substantial number of signs that may also be associated with autism. For example, Havdahl and colleagues [30] found that the presence of behavioural or emotional problems, as well as low IQ, had a marked influence on the discriminatory threshold of many commonly used diagnostic tools such as the ADOS, suggesting issues with specificity in a complex clinical setting. It would be informative to further explore which items, individual or combined, may be solely associated with autism and which items are also commonly observed in individuals with other conditions such as ADHD or intellectual disability.

Another possible explanation for the modest correlation between the ADOS total score and diagnostic certainty is that some clinicians may score ADOS items as present based on a range of qualitative expressions of a given sign [3], whereas only some of these expressions are recognized as autistic with high certainty. The distinction between different qualitative presentations is likely learned with experience and future research might investigate the association between qualitative variations in signs and diagnostic certainty.

### Correlations between certainty, head circumference, and IQ ratio

We found that individuals diagnosed with the highest certainty had a significantly larger normalized HC than those with lower certainty ratings for all three ADOS modules. Furthermore, 85% of individuals with the largest normalized HC, i.e., individuals within the top 2.5th percentile, were rated with the highest certainty versus 64% of individuals not meeting this criterion. This could indicate that either merely presenting with a larger head than commonly expected or having characteristics that are associated with having a larger HC in the autism population may influence the certainty of the clinician. Exploring associations between the normalized HC and other variables revealed small, but significant positive correlations with several items in the ADOS. Interestingly, certain items overlapped between modules; for example, Shared Enjoyment in Interaction across all three modules, as well as Imagination/Creativity and Reciprocal Social Communication in modules 2 and 3. Most of the significant correlations between HC and ADOS items were within the social interaction, play behavior, and communication domains. In addition, many of the ADOS items in modules 2 and 3 that correlated with HC were also associated with an increased likelihood of having the highest diagnostic certainty. For example in module 2, Shared Enjoyment in Interaction, Reciprocal Social Communication, Amount of Social Overtures, and Showing, which are all from the area of social communication and interaction, were significantly associated with certainty and were also found to be associated with HC. Although previous results on an association between autism symptom presentation and HC have been inconsistent, some studies indicate that particularly social symptoms may be associated with macrocephaly in autistic individuals [31, 32] while others link it to non-social atypicalities [33]. The largest correlation of HC was with the verbal to non-verbal ratio (*r* = -0.17), but only in module 2. Deutsch and Joseph [34] found a similar association between macrocephaly and verbal to nonverbal discrepancy in 2003 although with a larger correlation coefficient (*r* = -0.35). Interestingly, Joseph and colleagues [35] found that school age children with an IQ profile of higher non-verbal than verbal IQ had significantly higher autism symptomatology scores within the social interaction domain. Given the associations between diagnostic certainty, HC, social symptoms, and a low verbal/nonverbal IQ ratio, it would therefore be prudent to further explore whether these characteristics are part of a specific autism presentation that is recognized by clinicians with high certainty.

### Associations between certainty, language level, and age

Diagnostic certainty was associated with the age at assessment, as well as language level (ADOS module), with a significant interaction. A higher percentage of autistic children received the highest certainty rating when assessed with ADOS module 1 than those evaluated with modules 2 and 3, but the difference decreased with age. For those assessed with module 1 (no phrase speech), the percentage of high certainty was high regardless of age. For those assessed with module 2 (phrase but not fluent speech), diagnostic certainty was lower for children evaluated around three and six years old compared to children in age equivalent groups who were assessed with module 1. Interestingly, the percentage appeared to gradually reach the same high level as for module 1 for the children that are assessed at older ages. This likely reflects the fact that the absence of fluent speech becomes increasingly abnormal with age and, thus, those who are assessed with module 2 at older ages will likely be highly atypical compared to their age equivalent peers. A similar pattern was observed for those assessed with module 3, although the level of certainty was consistently slightly lower than for module 2, reflecting that a young child with highly developed language may be considered less likely to have autistic disorder.

### Association between certainty and other variables

We found several significant associations between certainty and IQ, as well as adaptive and externalizing behaviours, although not consistently across ADOS modules. Associations between diagnostic certainty and other variables have been explored in previous studies [8–12]. Negative associations between IQ and diagnostic certainty have been observed previously [9, 10, 12], consistent with our findings for those assessed with ADOS modules 2 and 3. Adaptive behaviour has been found to be negatively associated with certainty in some studies [10, 12] while others have found no association [8]. We found a negative association between certainty and externalizing behaviour among those assessed with ADOS module 3, while no association was found with internalizing behaviour. One previous study using data from the whole autism spectrum in the SSC found weak negative associations with both externalizing and internalizing behaviours [9], whereas another study found a positive association with internalizing behaviour and no association with externalizing behaviour [12]. Generally, the previous studies are difficult to directly compare to our results as they operationalized certainty differently. Some previous studies have considered the certainty of the clinician’s decision regardless of whether the decision was autism or no autism. Thus, those who clearly did not meet the criteria would have had a high certainty along with those who clearly did meet the criteria. Furthermore, previous studies investigated all children meeting the criteria for an autism *spectrum* diagnosis, whereas we limited our focus to the certainty of meeting the criteria for Autistic Disorder specifically. Certainty for a spectrum diagnosis may cover a broader range of signs, corresponding to the broad range of presentations that can fall within the autism spectrum, whereas certainty for an Autistic Disorder diagnosis may reflect recognition of a less variable presentation. As also mentioned by McDonnell and colleagues [9], sample characteristics may moderate associations between clinical factors and certainty. The fact that we stratified the sample based on language level (ADOS module), which the cited previous studies did not do, thus also makes direct comparison of the results more difficult.

### Limitations

The study focused on those diagnosed with Autistic Disorder, hypothesising that these individuals may be part of a subgroup corresponding to a particular prototype. The findings of this study, thus, do not describe certainty in a broader autism spectrum diagnosis. However, even the sample diagnosed specifically with Autistic Disorder contained variation, e.g. in terms of IQ, age at diagnosis, language level, and total ADOS score, and so might display some heterogeneity in terms of the factors that led to a clinician diagnosing them with higher or lower certainty. At the same time, the variation in the certainty rating among those diagnosed with Autistic Disorder was relatively low, with most individuals having certainty ratings close to the maximum value. This may have made it more difficult to detect associations between certainty and other variables. The heterogeneity of the sample is relevant for the interpretation of our results and may also have affected the magnitude of the identified effects. For example, an observed effect could be driven primarily by a smaller part of the population, but be diluted by other parts of the population that may have different mechanistic underpinnings. As such, the findings might be relevant for a small and potentially unknown subgroup, but not for most of the cohort represented in the SSC. Our finding demonstrating the correlation between HC and the verbal/nonverbal IQ ratio was only present in ADOS module 2. This highlights that it may be relevant to consider whether individuals can be stratified based on common features, such as language level or age at autism diagnosis, when investigating a heterogeneous autism population, [18] thereby making it more likely that the individuals have something in common. Analysing the whole population may result in the identification of a very small effect that is difficult to interpret. Phenotypic *a priori* stratification may decrease noise and make it more likely to identify larger effects that are relevant to the given subpopulation.

The demographic composition of the SSC may indicate a problem with representativeness, which can affect the interpretation of our findings. There was a high percentage of probands from families with a college degree, showing that the population studied had a higher level of education than that generally found in the adult US population [36]. The percentage of non-white groups was also low, particularly in the part of the sample assessed with module 3 that comprised only 2% African Americans. Predisposing factors of autism associated with, for example, race or the level of education may explain some of these discrepancies. However, it could also reflect a selection bias with certain demographic groups having better access to assessment facilities, thus impacting the generalizability of the findings from the SSC.

Clinicians’ diagnostic certainty is a subjective rating, and so it is expected to be associated with some degree of variability. For example, some clinicians may be certain more often than others, and different clinicians may not have the same understanding of what autism looks like depending on their clinical expertise and exposure to autism. Two clinicians thus may not report the same certainty rating if they were both to assess a given individual. Such differences in how certainty is rated introduce noise and would tend to decrease the size of the observable correlations between the certainty variable and the characteristics of the autistic individuals. Thus, our results likely do not show a *universal* pattern of how certainty correlates with clinical factors for every clinician, but rather represent an *averaged* picture across the participating clinicians and indicate those factors that are most associated with certainty. Furthermore, the clinicians contributing to the SSC cohort may not be representative of all clinicians performing autism assessments.

Finally, the study is an exploratory investigation of certainty for an Autistic Disorder diagnosis, and the findings should therefore be sought replicated in future studies.

## Conclusions

In this study we investigated clinical correlates of certainty for an Autistic Disorder diagnosis and found that certain ADOS items were more strongly associated with certainty than others, suggesting a difference in how much each item corresponds to what clinicians recognize as autistic. We also observed a positive association between certainty and normalized HC. Furthermore, we found normalized HC to correlate with some of the same ADOS items that were most highly associated with certainty. These items may be associated with a particular presentation of autism that also includes increased HC and which is recognized as DSM-IV autistic disorder with high certainty by clinicians.

## Data Availability

The data used in this study is from the Simons Simplex Collection and can be accessed by application through the Simons Foundation Autism Research Initiative (SFARI).

## Abbreviations

ADI: Autism Diagnostic Interview
ADOS: Autism Diagnostic Observation Schedule
CBCL: Child Behaviour Checklist
HC: Head circumference
SSC: Simons Simplex Collection
